# Further processing of soybean meal using thermomechanical and enzyme-facilitation modified secondary protein structure, *in-vitro* protein digestion kinetics and nutritive value in broiler chickens

**DOI:** 10.1016/j.psj.2025.106146

**Published:** 2025-11-21

**Authors:** Felix M. Njeri, Emily Kim, Youngji Rho, Mai Anh Ton Nu, Astrid Koppenol, Hagen Schulze, Matthew Nosworthy, Anna K. Shoveller, Lee-Anne Huber, Elijah G. Kiarie

**Affiliations:** a Department of Animal Biosciences, University of Guelph, ON, Canada; b Guelph Research and Development Centre, Agriculture and Agri-Food Canada, Guelph, ON, Canada; c AB Neo, a/s, Videbaek, Denmark; d Livalta, AB Agri Ltd, Peterborough, UK

**Keywords:** Broiler chickens, Amino acid and energy digestibility, Secondary protein structure, Thermomechanical and enzyme facilitated soybean meal processing

## Abstract

Further processing of protein feedstuffs, such as conventional soybean meal (**CSBM**), may improve their nutritive value. This study evaluated chemical attributes, *in-vitro* protein digestion kinetics, apparent (**AID**) and standardized (**SID**) ileal digestibility of amino acids (**AA**), and apparent metabolizable energy (**AME**) in CSBM subjected to thermomechanical and enzyme-facilitated processing (**PSBM**). For the *in vitro* digestion, samples were sequentially incubated in triplicate, with pepsin at pH 2.5 for 0 and 0.5 h (gizzard phase), then with pancreatin and bile extract for 0, 0.5, 1, 1.5, 2, 2.5 and 3 h (intestinal phase, pH 6.5). A total of 180 one-day-old Ross 708 male chicks were fed a commercial starter diet until day 13 (*n* = 6). On day 14, birds were assigned to one of three diets: a nitrogen-free diet (**NFD**), or test ingredients containing 20 % crude protein (CP) from either CSBM or PSBM as the sole source of AA. Titanium dioxide (0.5 %) was included as an indigestible marker. Excreta were collected between days 17 and 20, and ileal digesta on day 21. Fourier-transform infrared spectroscopy revealed structural shifts (*P* < 0.05) in PSBM compared to SBM, including increased β-sheets, emergence of 3_10-_helix, and reduced random coils and intramolecular aggregates. Compared to CSBM, PSBM had a higher proportion of fast-digestible protein (*P* < 0.001), representing a 21 % increase, and lower proportions of both slow-digestible (*P* = 0.001) and resistant protein fractions (*P* = 0.002). Compared to CSBM, PSBM had greater AID of CP, Lys, Met, and Thr (*P* = 0.001). The SID of Lys, His, and Val and total AA were greater in PSBM (*P* < 0.05) than CSBM. The PSBM tended to have greater AME than CSBM (*P* = 0.079). In conclusion, PSBM had modified secondary protein structure resulting in enhanced *in vitro* protein digestion kinetics profile, and improved digestibility of nutrients in broiler chickens

## Introduction

Conventional soybean meal (**CSBM**), a co-product of soybean oil extraction, acts as an important ingredient of animal nutrition, particularly in poultry diets. This is mainly due to its high crude protein (**CP**) content and balanced amino acid (**AA**) profile. The inclusion levels of CSBM into poultry diets are influenced by factors such as bird age and specific nutrients requirements of the birds and typical ranges are 25 % in young chicks to 30 to 40 % in older broilers and laying hens (Ravindran et al., 2014). However, the anti-nutritional factors (ANF) of CSBM, including lectins, glycinin and β-conglycinin, trypsin inhibitors, and oligosaccharides, limit its use by impeding the absorption of nutrients and the digestion of proteins (Erdaw et al., 2016; Kiarie et al., 2021). Young chicks are particularly vulnerable to these ANF due to their immature digestive system which leads to low endogenous enzyme production. This can result in poor growth, reduced feed conversion efficiency, and pancreatic hypertrophy (Tsementzis et al., 2025). Although older birds are better able to tolerate the ANF in soy products, they are never fully adapted. Additionally, structural barriers like intact cell walls and protein aggregations further restrict enzyme access, reducing digestibility across all ages (Wang et al., 2024). Thermomechanical-enzymatic processing (**TE**), that combines thermal and mechanical disruption with exogenous proteases, has emerged as a promising method to enhance nutrient release from CSBM (Ton Nu et al., 2020). This approach yields processed CSBM (**PSBM**) with enhanced protein solubility and digestibility by disrupting protein aggregates and cellular matrices.

Recent studies support TE processing potential. Ton Nu et al. (2020) and Njeri et al. (2025) demonstrated that PSBM improved *in vitro* CP digestion rates and increased **SID** of indispensable AA in pigs. In broilers, Colombino et al. (2023) demonstrated that PSBM enhanced ileal digestibility and intestinal villus height. Similar approaches combining physical and enzymatic fractionation have been shown to improve protein accessibility and reduce resistant β-sheet structures in CSBM (Salazar-Villanea et al., 2016).

A key limitation in current research lies in the incomplete understanding of how TE alters the protein structure of CSBM and how these structural modifications influence enzymatic accessibility and nutrient utilization in poultry. Excessive thermal exposure, though effective at denaturing ANF, can induce protein aggregation and the formation of stable β-sheet structures that resist enzymatic degradation (Carbonaro et al., 2012a; Zhang et al., 2022). In contrast, moderate heat treatments tend to promote the formation of disordered protein structures such as random coils, which are more readily digested by proteolytic enzymes (Salazar-Villanea et al., 2016; Wijethunga et al., 2024). Despite evidence from swine studies indicating that CSBM processed at 100–120°C enhances protein solubility and digestibility (González-Vega et al., 2011; Salazar-Villanea et al., 2016), equivalent data in poultry remain scarce. Moreover, the structural consequences of TE are rarely assessed using direct analytical techniques in poultry-specific studies. Advanced tools such as Fourier-transform infrared (FT-IR) spectroscopy, which can reveal changes in protein secondary structure, particularly in α-helix to β-sheet and random coil content, are underutilized in poultry nutrition research (Doiron and Yu, 2017; Peng et al., 2014; Pfeuti et al., 2020). Consequently, the relationship between thermally induced structural transitions and digestibility outcomes remains speculative, leading to largely empirical approaches in the optimization of PSBM for poultry diets (Rodriguez-Espinosa et al., 2025).

This study investigated the effects of TE processing on CSBM by quantifying changes in secondary protein structure using FT-IR spectroscopy, kinetics of soluble nitrogen (**SN**) release during *in vitro* digestion, and evaluating secondary protein structural modifications, digestion kinetics, AID and SID of CP and AA, and AME in broilers. We hypothesized that TE processing induced specific, measurable changes in protein conformation, such as a reduction in β-sheet content and an increase in random coil content, which enhanced SN release during digestion and improved ileal nutrient digestibility compared to CSBM.

## Materials and methods

### Experimental procedures

The use of animals was approved by the University of Guelph Animal Ethics Committee and complied with the Canadian Code of Practice for the Care and Use of Animals for Scientific Purposes (CCAC, 2009).

#### Materials

The study had two independent experiments: Exp. 1 for *in-vitro* digestion simulation and Exp. 2 for the determination of the nutritive value in broiler chickens. The experiments were conducted at different times and necessitated the procurement of samples at various time points. In Exp. 1, the CSBM sample was procured from Ontario-based vendors through the Arkell Feed Mill (Guelph, ON, Canada). The PSBM sample used in Exp. 1 was a commercially available product (AlphaSoy 530, AB Neo, Videbaek, Denmark; Table 1). Pepsin (P7000, ≥250 units/mg solid) and pancreatin (P1750, 4 × USP specifications) were purchased from Sigma-Aldrich Canada Ltd. Porcine bile extract (CAS 8008-63-7) was obtained from Santa Cruz Biotechnology. The CSBM sample used in Exp. 2 was the unprocessed starting material used to produce PSBM and was obtained from the same manufacturer (AB Neo, Videbaek, Denmark). The details of processing CSBM to generate PSBM was previously described (Ton Nu et al., 2020). Briefly, PSBM was produced using a four-stage thermo-mechanical process with enzymatic assistance. Raw SBM was first ground with a hammer mill to achieve a mean particle size of approximately 500 μm. The ground SBM was conditioned with steam and water, and a blend of proteases was added as a processing aid. These extrudates were then re-milled with a hammer mill to obtain a mean particle size of approximately 400 μm. For experimental comparison, unprocessed SBM was ground through a 3-mm screen, yielding an average particle size of 564 μm, while processed TES averaged 442 μm.

**Table 1:** Analyzed ingredient composition presented on a dry matter basis.

	Exp 1		Exp 2	
Item	CSBM^1^	PSBM^2^	CSBM^3^	PSBM
Crude protein (CP), %	51.4	57.5	48.88	49.64
Gross energy, kcal/kg	4,512	4,535	4,744	4,943
Acid detergent fiber, %	3.65	4.54	9.62	8.94
Neutral detergent fiber, %	10.07	12.10	14.38	14.01
Arg, %	3.62	3.96	4.75	4.32
His, %	1.34	1.40	1.25	1.15
Ile, %	2.01	2.53	1.93	1.94
Leu, %	3.64	4.25	3.22	3.19
Lys, %	2.59	2.91	2.47	2.48
Met, %	0.93	0.93	0.65	0.68
Phe, %	2.78	3.16	2.46	2.41
Thr, %	1.92	2.06	1.64	1.64
Val, %	2.06	2.49	2.19	2.10
Ala, %	1.96	2.26	1.81	1.78
Asp, %	5.27	6.4	4.58	4.54
Cys, %	0.82	0.84	0.74	0.74
Glu, %	8.63	10.09	7.59	7.50
Gly, %	2.17	2.42	2.16	1.96
Pro, %	2.48	2.84	2.54	2.30
Ser, %	2.65	2.97	2.24	2.21

1 Conventional soybean meal (SBM) sourced from a different region from that of PSBM.

2 Further processed SBM through combination of thermomechanical and protease.

3 Was the starting material for Exp 2 PSBM production (Table 2).

#### Experiment 1

This study evaluated *in vitro* nitrogen solubilization in CSBM (CP, 51.4 % CP on DM basis) and PSBM (CP, 57.5 % DM; Table 1). A modified *in vitro* protocol as described previously (Boisen and Fernández, 1995; Njeri et al., 2025) was used. Aliquots of 500 mg (CP basis) of each test ingredient were weighed into 50 mL centrifuge tubes. During the gastric phase simulation, 25 mL of 0.1 M phosphate buffer (pH 6.0) and 2 mL of pepsin solution (27 mg/mL) were added. The mixture pH was modified to 2.5 using predetermined volumes of 1 M HCl, as identified in preliminary tests. Three randomly selected replicates underwent pH verification, with adjustments applied to all samples if discrepancies arose. However, discrepancies were infrequent, as a pretest was conducted on the same day before the actual experiment Triplicate samples underwent sequential incubation in a temperature-controlled shaker (New Brunswick™ Innova® 43 Series) at 39°C and 200 rpm for intervals of 0 and 0.5 h. For intestinal modeling, gastric-digested samples were mixed with 10 mL of 0.2 M phosphate buffer (pH 6.8), 2 mL of pancreatin (100 mg/mL), and 2 mL of bile salts (117 mg/mL). All mixtures were neutralized to pH 6.5 using 0.1 M NaOH and incubated for 0, 0.5, 1, 1.5, 2, 2.5, and 3h . Post-incubation samples were flash-frozen in liquid nitrogen, later on thawed at 4°C, and centrifuged (2,910 × g, 10 min). The supernatant was transferred to fresh 50 mL tubes and stored frozen at −20°C for subsequent nitrogen analysis, while the pellet underwent lyophilization.

#### Experiment 2

A nitrogen free diet (NFD), corn starch-based diet was formulated (Table 2). The NFD was meant to allow estimation of endogenous nitrogen losses for the calculation of SID of amino acids (Stein et al., 2007). Two experimental diets were formulated using either CSBM or PSBM as the sole source of AA and were designed to contain ∼20 % CP with the ratio of corn starch-to-sucrose-to-soy oil (the sole sources of energy in NFD) maintained constant to allow determination of nitrogen corrected apparent metabolizable energy (**AMEn**) in test ingredients by the substitution method (Woyengo et al., 2010). All then test diets contained TiO_2_ (0.50 %) as an indigestible marker and were fed as mash (Table 2). The analyzed chemical composition of diets are presented in Table 3.

**Table 2:** Diet composition, as fed (Exp. 2).

	NFD^2^	CSBM	PSBM
Corn starch	76.78	45.18	49.79
PSBM	-	-	37.33
SBM	-	43.15	-
Sucrose	8.25	4.85	5.35
Cellulose	5.00	-	-
Soy oil	2.5	1.47	1.62
Monocalcium phosphate	2.32	1.9	2.32
Limestone Fine	1.29	1.2	1.34
Vitamin and trace minerals premix^1^	1.00	1.00	1.00
Salt	0.38	0.37	0.37
Magnesium oxide	0.17	-	-
Sodium bicarbonate	0.04	-	-
Potassium carbonate	1.77	0.38	0.38
Titanium dioxide	0.50	0.50	0.50
Calculated provisions			
AME poultry	2,872	2,713	2,862
Crude protein	-	20	20
Crude fat	2.4	1.41	2.49
Calcium	0.88	0.9	0.9
Total phosphorous	0.49	0.67	0.49
Available phosphorous	0.43	0.43	0.43
Sodium	0.16	0.16	0.16
Chloride	0.23	0.23	0.23
Magnesium	0.10	0.15	0.13
Potassium	1.00	1.00	1.00

1 Provided per kilogram of diet: vitamin A, 8800.0 IU; vitamin D3, 3300.0 IU; vitamin E, 40.0 IU; vitamin B12, 12.0 mg; vitamin K3, 3.3 mg; niacin, 50.0 mg; choline, 1200.0 mg; folic acid, 1.0 mg; biotin, 0.22 mg; pyridoxine, 3.3 mg; thiamine, 4.0 mg; calcium pantothenic acid, 15.0 mg; riboflavin, 8.0 mg; manganese, 70.0 mg; zinc, 70.0 mg; iron, 60.0 mg; iodine, 1.0 mg; copper, 10 mg; and selenium, 0.3 mg.

2 nitrogen free diet.

**Table 3:** Analyzed composition of experimental diets, as fed (Exp. 2).

Item	NFD	CSBM	PSBM
Dry matter, %	92.8	90.7	92.1
NDF, %	-	9.67	8.99
ADF, %	-	3.78	3.47
Gross energy, kcal/kg	3,529	3,678	3,717
Crude protein, %	0.395	22.4	20.51
Arg, %	0.045	1.97	1.72
His, %	0.004	0.54	0.43
Ile, %	0.014	0.83	0.72
Leu, %	0.019	1.39	1.19
Lys, %	0.012	1.07	0.93
Met, %	0.022	0.25	0.28
Phe, %	0.026	1.06	0.90
Thr, %	0.027	0.71	0.61
Val, %	0.016	0.95	0.78
Ala, %	0.018	0.78	0.66
Asp, %	0.016	1.98	1.69
Cys, %	0.025	0.38	0.31
Glu, %	0.053	3.27	2.80
Gly, %	0.008	0.93	0.73
Pro, %	0.005	1.10	0.86
Ser, %	0.031	0.97	0.82
Tyr, %	0.009	0.82	0.65

#### Animals, procedures and sample collection

A total of 180-day old male broiler chicks (Ross 708) were allocated to 12 identical floor pens (15 chicks per pen) based on body weight (BW) and were fed *ad libitum* with a commercial broiler starter diet up to d 13. On d 14, the birds were distributed in cages based on BW (10 chicks/cage; *n* = 6) and allocated to 1 of the 3 experimental diets. The cages were placed in environmentally controlled rooms, where the temperature was maintained at 29°C until day 13 and then gradually reduced to 24°C by day 21. The birds received 20 h of light daily and had free access to water throughout the experimental period. Between d 17 and 20 post-hatching, pooled excreta samples were collected per cage for the determination of nutrient retention and AMEn. On day 20, all the chicks were euthanized by cervical dislocation for the collection of ileal digesta. Digesta from birds within a cage were pooled, resulting in 6 samples per dietary treatment, and frozen immediately after collection (Adedokun et al., 2007).

#### Sample processing and chemical analyses

For Exp. 1, samples of CSBM and PSBM were analyzed for secondary protein structure using Attenuated Total Reflectance Fourier-Transform Infrared (ATR-FTIR) Spectroscopy (Pfeuti et al., 2020). The spectra were acquired using a Bruker Alpha II spectrometer equipped with a diamond crystal plate. Solid samples were prepared by applying sufficient powder to coat the crystal surface with a uniform layer (approximately 1 mm thick), and the material was compressed using the integrated pressure arm. Before analysis, the diamond crystal and anvil tip were cleaned by wiping with a Kimwipe moistened with alcohol, followed by thorough drying. A background spectrum was measured to account for atmospheric contributions, and sample spectra were recorded over the 4000–400 cm⁻¹ range (32 scans, 2 cm⁻¹ resolution). The data were baseline-corrected and smoothed using Savitzky–Golay filtering (9 points) in OPUS software prior to deconvolution.

For secondary structure analysis, the Amide I region (1600–1700 cm⁻¹) was resolved using Fourier self-deconvolution (FSD) followed by Gaussian curve fitting in PeakFit software v4.12 (SeaSolve Software Inc., California, USA). The deconvoluted spectra were then fitted with Gaussian functions to quantify secondary structural components based on established protocols. Peaks found at 1600–1605 cm⁻¹ were identified as sidechain vibrations. A peak at 1618 cm⁻¹ corresponded to intermolecular antiparallel β-sheet aggregates was identified (literature range: 1610–1620 cm⁻¹). Peaks between 1620 and 1640 cm⁻¹ were attributed to parallel β-sheet. Peaks at 1640–1650 and 1660–1670 cm⁻¹ were assigned to random coil. Peaks between 1650 and 1660 cm⁻¹ were identified as α-helix. Peaks between 1670 and 1680 cm⁻¹ were associated with antiparallel β-sheet. Peaks in the ranges 1660–1670 and 1680–1700 cm⁻¹ were assigned to β-turns (Table 4). These assignments align with established correlations for protein secondary structures derived from Fourier self-deconvolution and Gaussian fitting. Each sample was analyzed in triplicate to ensure reproducibility, with the final peak positions and areas averaged across replicates. Spectral graphs were visualized and annotated using OriginPro 2025 Learning Edition (Origin Lab Corporation, Northampton, MA, USA).

**Table 4:** ATR-FTIR Amide I band assignments for protein secondary structures.

Wavenumber (cm⁻¹)	Secondary structure	Structural interpretation	References
1600–1620	Aggregated β-sheet	Strongly hydrogen-bonded strands; often intermolecular or denatured β-sheets	Cobb et al., 2020; Kong and Yu, 2007b; Long et al., 2015
1621–1640	Intramolecular β-sheet	Regular β-strands; moderate internal hydrogen bonding	Barth, 2007; Long et al., 2015
1641–1650	Random coil	Disordered/unstructured polypeptide backbone	Barth, 2007; Long et al., 2015
1651–1658	α-Helix	Canonical α-helical structure; stable intrachain hydrogen bonding	Barth, 2007; Kong and Yu, 2007b
1659–1663	3₁₀-Helix	Tighter helical turns; overlaps high-frequency end of α-helix region	Cobb et al., 2020; Kong and Yu, 2007b; Zhang et al., 2022a
1664–1670	β-Turn	Reverse turns or flexible loop segments (Type I/II)	Kong and Yu, 2007b; Long et al., 2015; Vanga et al., 2016; Zhang et al., 2022a
1671–1680	Antiparallel β-sheet	Extended strands aligned in opposite orientation	Long et al., 2015; Vanga et al., 2016
1681–1690	β-Turn	Loop or terminal structures with variable hydrogen bonding	Barth, 2007; Kong and Yu, 2007b
1691–1700	High-frequency β-sheet	Aggregated or antiparallel strands with strong dipole–dipole interactions	Long et al., 2015; Vanga et al., 2016

Samples of the soy products, experimental diets, air dried excreta (60 °C until constant weight) and freeze-dried ileal contents were finely ground using a coffee grinder (CBG5 Smart Grind; Applica Consumer Products, Inc., Shelton, CT). Ingredients, diets, and excreta samples were analyzed for DM, gross energy (**GE**), neutral detergent fiber (NDF), nitrogen, crude fat, and titanium. Method 930.15 AOAC (2005) was used for DM analyses. The gross energy (GE) was determined using an adiabatic bomb calorimeter with benzoic acid as a standard (IKA Calorimeter System C 6000; IKA Works, Wilmington). The NDF was determined using ANKOM 200 fiber analyzer (ANKOM Technology, Fairport, NY) according to van Soest et al. (1991). Nitrogen was determined using the LECO machine (LECO Corporation, St. Joseph, MI) using method 968.06 (AOAC, 2005), and CP values were derived by multiplying the N value by 6.25. Crude fat content was determined using ANKOM XT 20 Extractor (ANKOM Technology, Fairport, NY). The titanium content was determined according to Myers et al. (2004). The soy samples, diets and ileal digesta were further oxidized, hydrolyzed and analyzed to quantify AA concentrations using ultra-performance liquid chromatography (UPLC; Waters Corporation, Milford, MA, USA) (Thanabalan et al., 2021). Tryptophan was not determined.

#### Calculations and statistical analyses

Intestinal CP solubilization (CPS) was classified into three fractions: CP_fast_, CP_slow_, and CP_resistant_, representing protein solubilized within the first 0.5 h, between 0.5 and 2.5 h, and the portion remaining undigested after 2.5 h, respectively. The CP_resistant_ fraction was calculated as:

CP_resistant_ = 100 – (CP_fast_ + CP_slow_). To model CPS kinetics during the intestinal phase, the Gompertz function was applied using the PROC MODEL procedure in SAS (SAS Institute Inc., Cary, NC), following the method outlined by Njeri et al. (2024), Njeri et al. (2024):

$$\%\;\text{solubilized}\;\text{CP}\;\text{of}\;\text{total}\;\text{CP}=A\;\times \;\left(\frac{A}{W_{o}}\right)exp\left(-e\;\times K_{U}\;\times \;\text{Time}\right)$$

In this equation, A denotes the maximum proportion of nitrogen solubilized (as % of total CP in undigested samples) at the asymptote. Wo refers to the CP solubilized at the beginning (0 h) of the intestinal phase, equivalent to nitrogen solubilized at the end of the gastric phase (1.5 h). Ku represents the maximum rate of nitrogen solubilization (h⁻¹) at the inflection point, and Ti corresponds to the time at which this inflection occurs.

The **AID** of AA and retention (**AR**) of components and AMEn were calculated according to Woyengo et al., 2010. Basal endogenous losses of CP and AA (**ENL**) were calculated from digesta of birds fed NFD, using the approach described by Stein et al. (2007). The **SID** of CP and AA were then determined by adjusting the AID values for ENL.

Data analysis was conducted using the PROC GLIMMIX procedure in SAS (SAS Institute Inc., Cary, NC). For *in vitro* gastric and ileal digestion, the model accounted for the interaction between protein source and time. Similarly, for CP classification and protein digestion kinetics parameters, processing method was considered a fixed effect. The average values of these kinetics’ parameters were used to estimate and visualize the *in vitro* protein digestion kinetics equations. In Experiment 2, the model also included protein source as the fixed effect, with cage as the experimental unit. Statistical significance was established at *P* < 0.05, while a trend was indicated when 0.05 ≤ *P* < 0.10. Tukey's test was used to compare significant means.

## Results

For Exp. 1, CSBM from different origin was used resulting in different AA composition. PSBM had numerically higher concentration of CP (57.5 vs. 51.4 % on DM basis) than CSBM but had 28.3 % lower soluble CP (Table 1). CSBM had consistently higher Thr, Ile, and Val in Exp 1, but differences narrowed or reversed in Exp 2. Lys, Met, Cys showed minor or negligible differences across both experiments. The ingredients used during the *in vivo* study had comparable chemical composition.

**Fig. 1:**
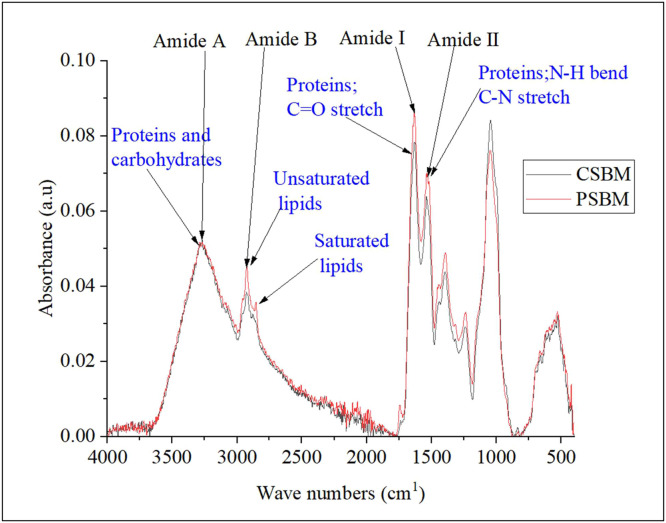
ATR−FTIR spectrum of CSBM and PSBM acquired with the Bruker Alpha II spectrometer.

**Fig. 2:**
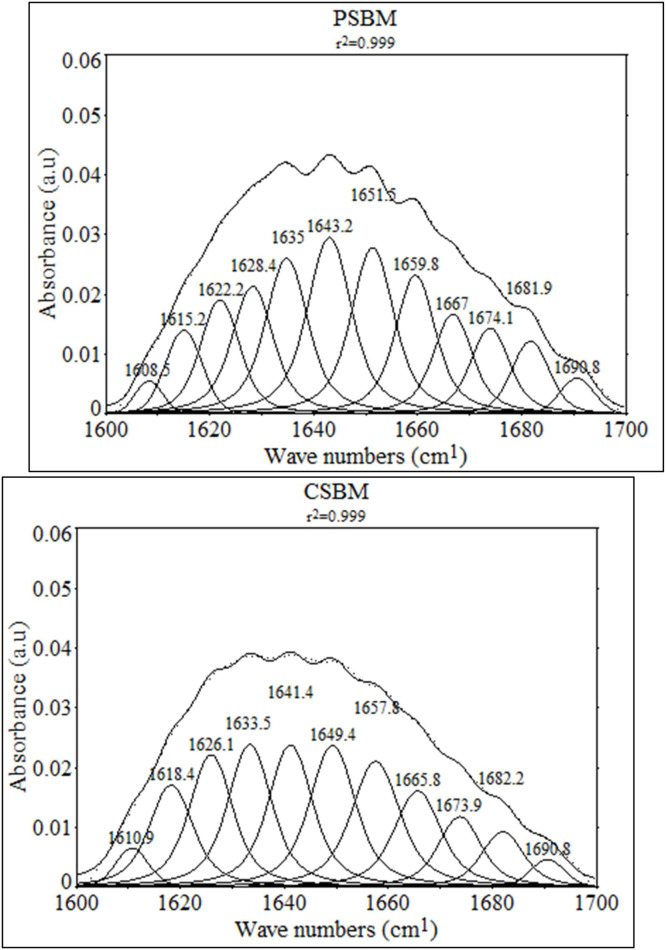
Deconvoluted amide I region (1600 – 1700 cm^1^) ATR−FTIR spectrum of CSBM and PSBM acquired with the Brucker Alpha II spectrometer.

The ATR-FTIR for test ingredients are presented in Fig. 1 while the deconvoluted amide I region are presented in Table 5 and Fig. 2. Deconvolution of the Amide I region revealed that TE resulted in a reduction of β-sheet aggregates from 12.11 to 8.52 %, an increase of ordered β-sheet from 36.16 to 40.25 %, and about 11.7 % more α-helix content compared to CSBM sample (*P* < 0.001). Additionally, PSBM had some of its secondary structures converted into 3₁₀_Helix, with this type of helices not being detected in CSBM. Moreover, PSBM exhibited a reduction of random coil content from 27.6 to 13.6 % (*P* < 0.001) while the β-turns conformations increased from 12.8 to 13.6 (*P* < 0.001) as compared to CSBM. On the other hand, the α-helix/β-sheet ratio, reflecting structural order, increased in PSBM from 23.8 to 49.3 % compared to CSBM. The CSBM had higher total amide I than PSBM (*P* < 0.001).

**Table 5:** Secondary structures of deconvoluted Amide I area (Exp. 1).

Item,%	CSBM	PSBM	SEM	P-value
β-Sheet aggregates	12.11	8.52	0.31	<0.001
β-Sheet	36.16	40.25	0.28	<0.001
Total β-sheet	48.27	48.78	0.33	0.194
Random coil	27.60	13.57	0.69	<0.001
α-Helix	11.35	12.68	1.33	<0.001
3₁₀_Helix	nd	11.38	-	-
Total helices	11.35	24.07	0.37	<0.001
β-Turn	12.78	13.59	0.84	0.393
α-Helix/β-Sheet	23.51	49.34	0.74	<0.001
Total Amide I area	3.05	2.6	0.04	<0.001

nd = not detected.

The *in vitro* N solubilization by pepsin and pancreatin is summarized in Table 6. In the gastric phase, the interaction between time and test ingredients on N solubility was such that at 0h it was lower in CSBM (*P* = 0.001) than in PSBM. However, at 0.5 h, PSBM had a 20.4 % lower solubility than CSBM, (*P* = 0.036). Although, shifting the pH to 6.5 during intestinal phase at 0 h increased the soluble N in PSBM by 48.1 % relative to CSBM (*P* = 0.007). Between 0.5 h and 1 h, PSBM exhibited higher N solubility than CSBM (*P* = 0.002). There were no differences between ingredients on N solubility at 1.5 (*P* = 0.794) and 2 h (*P* = 0.876). However, at 2.5 h, PSBM had greater N solubility than CSBM (*P* = 0.007). At 3 h, N solubility was similar between PSBM and CSBM (*P* = 0.290).

**Table 6:** *In vitro* gastric and small intestine digestion of CP in CSBM and PSBM (Exp. 1).

Item %		Protein source		SEM	P-values	
	Time, h	CSBM^1^	PSBM^2^		Protein source	P*Time
Gastric phase						
	0.0	6.03	15.33	1.63	0.001	<0.001
	0.5	30.12	23.96	1.98	0.036	
Intestinal phase						
	0.0	37.27	55.20	3.51	0.007	<0.001
	0.5	65.07	78.65	0.99	0.002	
	1.0	80.54	84.55	0.40	0.002	
	1.5	84.22	84.46	0.85	0.794	
	2.0	85.09	85.32	1.41	0.876	
	2.5	88.52	92.74	0.55	0.007	
	3.0	91.62	89.81	2.35	0.290	

1 Conventional soybean meal (SBM).

2 Further processed SBM through combination of thermomechanical and protease.

**Fig. 3:**
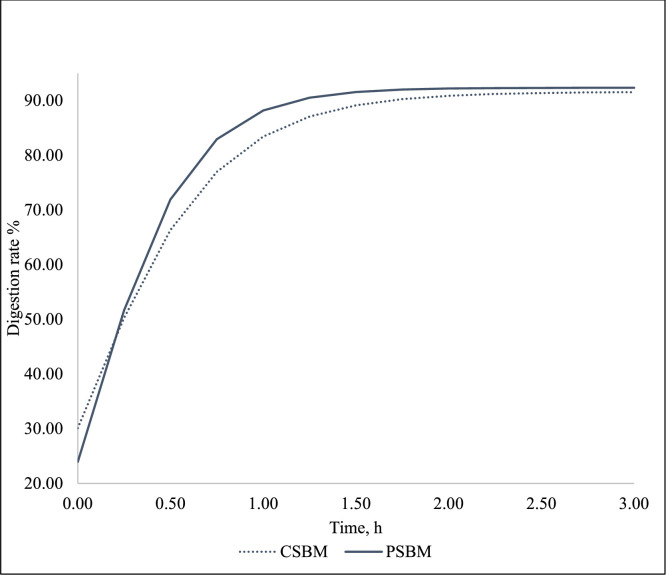
Calculated *in vitro* protein digestion kinetics equation in the small intestine for CSBM [*Y* = 91.62 × (91.62/30.12) exp(-e∗0.91∗Time))] and PSBM [*Y* = 92.38 × (92.38/23.96)exp(-e∗1.24∗Time))] (Exp. 1).

The small intestine CP (CP = solubilized *N* × 6.25) digestion kinetics from Exp 1 are presented in Table 6 and Fig 3. Differences in crude protein digestion kinetics between the two soybean meal sources were observed. Compared to CSBM, PSBM had a higher proportion of fast-digestible protein (*P* < 0.001), representing a 21 % increase, and lower proportions of both slow-digestible (*P* = 0.001) and resistant protein fractions (*P* = 0.002). On a per-kilogram basis, fast-digestible protein was 35 % higher in PSBM (*P* = 0.008), while slow and resistant fractions were lower by 33 % (*P* < 0.001) and 29 % (*P* = 0.002), respectively. PSBM also showed a lower initial digestible protein fraction (W_o_; *P* = 0.019), a higher digestion rate (Ku; *P* = 0.033), and no differences in the estimated maximum digestibility (A; *P* = 0.698) or the time to peak digestion (Ti; *P* = 0.933) (Table 7).

**Table 7:** *In vitro* kinetics of small intestine of digestion of CP in CSBM and PSBM (Exp.1).

Item	Protein source		SEM	P-value
	CSBM	PSBM		
Protein classification based on digestion rate				
Crude protein_Fast %_	65.07	78.65	0.99	<0.001
Crude protein_Slow %_	23.46	14.09	3.85	0.001
Crude protein_Resistant %_	11.47	7.26	1.70	0.002
g/kg				
Crude protein_Fast_ g/kg	334.46	452.21	19.63	0.008
Crude protein_Slow_ g/kg	120.56	81.02	21.57	<0.001
Crude protein_Resistant_ g/kg	58.97	41.77	8.74	0.002
Kinetics parameter estimates1				
Wo % digested CP of total CP	30.12	23.96	1.93	0.019
A % digested CP of total CP	91.62	92.38	1.82	0.698
Ku % digested CP of total CP/h	0.91	1.24	0.09	0.033
Ti, h	−0.40	−0.40	0.04	0.933

^1^The rate of protein digestion was described by a Gompertz model, which incorporation parameters such as maximum digested protein (A), initial protein content (W_o_) in the small intestine, maximum digestion rate (K_u_), and the time at which the digestion rate peaks (Ti).: % Digested CP (of total CP) = *A* × (A/W_o_) exp(−*e* × K_u_ × Time).

The AID and SID of CP and AA for CSBM and PSBM fed to broiler chickens are presented in Table 8, Table 9. The PSBM had greater AID of CP than CSBM (88.5 vs. 86.2 %; *P* = 0.001). The AID of mean indispensable AA was greater for PSBM than CSBM (87.9 vs. 86.1 %; *P* = 0.004). However, the AID of Met was lower in PSBM (*P* = 0.046) than CSBM. The mean AID of dispensable amino acids and the SID of crude protein were significantly higher in PSBM than in CSBM (*P* < 0.05; Table 8, Table 9). Among indispensable AA, PSBM had higher SID of His (*P* = 0.002), Leu (*P* = 0.018), Phe (*P* = 0.029), and Val (*P* = 0.004) than CSBM (Table 9). The SID of mean indispensable and dispensable AA was greater (*P* = 0.049) in PSBM than CSBM.

**Table 8:** Apparent ileal digestibility of CP and AA in SBM and PSBM fed to 21-day-old broiler chickens (Exp. 2).

Item	CSBM	PSBM	SEM	P-value
Crude protein	86.2	88.5	0.376	0.001
Indispensable AA				
Arg	85.6	85.7	0.68	0.969
His	88.6	91.7	0.45	0.001
Ile	84.6	86.1	0.32	0.007
Leu	85.3	87.4	0.40	0.004
Lys	89.8	91.8	0.54	0.026
Met	91.5	90.3	0.37	0.046
Phe	84.5	86.4	0.39	0.008
Thr	81.9	85.3	0.84	0.017
Val	83.4	86.2	0.34	0
Mean indispensable AA	86.1	87.9	0.33	0.004
Dispensable AA				
Ala	86.3	89.2	0.55	0.004
Asp	86.6	89.3	0.60	0.009
Cys	75.8	74.6	1.06	0.441
Glu	90.2	92.5	0.42	0.003
Gly	82.9	87.2	0.66	0.001
Pro	84.9	89.5	0.60	0
Ser	86.2	88.3	0.56	0.026
Tyr	86	87.8	0.35	0.005
Mean dispensable AA	84.9	87.3	0.52	0.008

**Table 9:** Coefficients of standardized ileal digestibility of CP and AA % in SBM and PSBM fed to 21-day-old broiler chickens (Exp. 2).

	CSBM	PSBM	SEM	p-value
Crude protein	89.6	91.6	0.376	0.004
Indispensable AA				
Arg	87.3	87.1	0.68	0.846
His	91.2	93.7	0.45	0.002
Ile	88.3	89.3	0.32	0.052
Leu	88.5	90.1	0.40	0.018
Lys	92.5	94.1	0.54	0.060
Met	94.3	93.4	0.37	0.118
Phe	87.0	88.4	0.39	0.029
Thr	90.7	92.8	0.84	0.109
Val	88.5	90.3	0.34	0.004
Mean indispensable AA	89.8	91.0	0.33	0.024
Dispensable AA				
Ala	91.1	93.2	0.55	0.022
Asp	89.7	92.0	0.60	0.023
Cys	86.1	84.2	1.06	0.236
Glu	92.8	94.7	0.42	0.011
Gly	87.5	90.8	0.66	0.005
Pro	90.5	93.7	0.60	0.003
Ser	92.7	93.7	0.56	0.217
Tyr	88.7	89.9	0.35	0.033
Mean dispensable AA	89.9	91.5	0.52	0.049
Total AA	87.4	89.4	0.35	0.029

PSBM-Thermomechanical and enzyme-facilitated processed soybean meal.

SBM-soybean meal.

The AR of components and AME in 21-day-old broiler chickens fed CSBM and PSBM are presented in Table 10. There were no differences between PSBM and CSBM on AR of DM, organic matter, NDF, GE and CP (*P* > 0.05). The AME tended to be 1.9 % higher in PSBM (3,256 vs. 3,196 kcal/kg DM, *P* = 0.079) but AMEn was not different (3,038 vs. 3,005 kcal/kg DM, *P* = 0.263).

**Table 10:** Apparent retention of components and AME in CSBM and PSBM fed to 21-day-old broiler chickens (Exp. 2).

	CSBM^1^	PSBM^2^	SEM	P-value
Dry matter%	73.9	74.3	0.71	0.706
Organic matter %	77.3	78.3	0.58	0.246
Neutral detergent fiber %	28.0	20.9	3.08	0.140
Gross energy %	79.1	80.3	0.53	0.167
Crude protein %	65.0	67.1	1.06	0.175
AME, kcal/kg DM	3,196	3,256	21.64	0.079
AMEn, kcal/kg DM	3,005	3,038	19.38	0.263

1 Conventional soybean meal (SBM).

2 Further processed SBM through combination of thermomechanical and protease.

## Discussion

The differences in chemical composition observed between CSBM and PSBM largely reflected the inherent variation in the raw soybean meal sources rather than the effects of TE processing (Table 1). In Exp. 1, CSBM and PSBM were sourced from different regions, while in Exp. 2, CSBM served as the starting material for the processed batch. Accordingly, variations in the concentrations of CP, AA, and fiber fractions are attributable to differences in the chemical characteristics of the base material. This interpretation is consistent with published work showing that the nutrient composition of CSBM is strongly influenced by region of cultivation, genotype, and growing conditions, with notable differences even under similar processing conditions (Aguirre et al., 2022; Lagos and Stein, 2017; Ravindran et al., 2014). In this context, the compositional data in this context should be interpreted with caution and not be used as a proxy for evaluating the effects of processing alone.

Secondary protein structure plays a crucial role in regulating solubility, enzymatic accessibility, and overall protein digestibility in feedstuffs (Kong and Yu, 2007b; Yang et al., 2016). Thermal processing combined with enzymatic hydrolysis interventions often disrupt hydrogen bonding, leading to either unfolding or controlled reorganization of structures such as β-sheets, α-helices, and random coils (Yanqing et al., 2022). In the present study, TE processing of CSBM induced a reduction in β-sheet aggregates while modestly increasing organized β-sheets and markedly enhancing total helical structures. The emergence of 3₁₀-helices in PSBM, which are compact and flexible transitional structures stabilized by *i* + 3 hydrogen bonds is particularly noteworthy (Tonlolo and Benedetti, 1991). Similar structural transitions have continued to be documented following mild thermal, hydrostatic pressure, or electric field processing of food proteins, where either random coils, α-helices or β-turns convert into 3₁₀-helices enhancing molecular flexibility without extensive denaturation (Cepero-Betancourt et al., 2020; Vanga et al., 2015; Wang et al., 2020). Such intermediate structures improve enzymatic accessibility by softening the tertiary architecture while retaining key intramolecular stabilizing interactions. These findings support the growing recognition that controlled structural reorganization, rather than wholesale unfolding, is critical for optimizing protein functionality post-processing (Salazar-Villanea et al., 2016). In the current study, the increase in the α-helix/β-sheet ratio and the reduction in the total Amide I area further indicated selective disruption of rigid hydrophobic β-sheet, creating a more soluble and enzyme-accessible protein substrate after TE of SBM.

*In vitro* digestion confirmed that PSBM exhibited higher initial nitrogen solubility under gastric conditions, likely due to increased exposure of peptide bonds. The high soluble N observed at the 0 h of gastric phase could be due processing induced generation of low-molecular-weight soluble peptides (Bing et al., 2024; Tacias-Pascacio et al., 2020). However, a transient decline in solubility during the gastric phase was observed, probably due to secondary aggregation of hydrophobic peptide fragments in the acidic environment (Barth, 2007) . This aggregation was reversible during the intestinal phase as pH shifted, restoring solubility and promoting continued hydrolysis. The distinct intestinal digestion patterns of CSBM and PSBM further reflected their secondary structure profiles. Although PSBM exhibited slightly higher total β-sheet content, these were predominantly intramolecular and embedded within partially flexible α/β-domain structures, as indicated by its higher α-helix/β-sheet ratio and lower total Amide I area (Barth, 2007). In contrast, CSBM contained a greater proportion of intermolecular β-sheet aggregates stabilized by dense hydrogen bonding and hydrophobic clustering (Doglia et al., 2008; Yu, 2005). This aggregation initially restricts enzymatic hydrolysis but progressively unfolds under prolonged intestinal conditions through deprotonation and increased charge repulsion (Carbonaro et al., 2012, 2008; Carbonaro and Nucara, 2010). This gradual structural relaxation exposed previously inaccessible cleavage sites, allowing CSBM to reduce the digestibility gap with PSBM. Meanwhile, PSBM, although rapidly digested initially, experienced slight secondary aggregation of hydrophobic peptides at later stages, modestly reducing nitrogen solubility. Collectively, these observations highlight that optimizing secondary structure, reducing excessive β-sheets while promoting helical flexibility, is critical for enhancing intestinal digestibility of processed plant proteins (Barth, 2000; Murayama and Tomida, 2004).

The *in vivo* SID data corroborated the *in vitro* findings, indicating that processing of PSBM improved CP digestibility in broilers. Specifically, *in vitro* assays showed a 4.5 % increase in N solubility for PSBM over CSBM at the 2.5-hour intestinal phase, closely aligning with *a* + 2.2 % improvement in SID of CP. Although gastric/proximal digesta were not collected, the *in vitro* pepsin-solubilization assay indicated an 18 % increase in pepsin-labile nitrogen. This finding, aligned with terminal ileal *in vivo* outcomes, and is consistent with a contribution of early-phase structural changes to improved digestibility; therefore, this is presented as a mechanistic hypothesis rather than direct validation. These changes likely include partial denaturation of glycinin and β-conglycinin and thermal or enzymatic inactivation of ANF such as trypsin inhibitors and lectins (Ton Nu et al., 2020) . The slight improvements in protein utilization observed with PSBM compared to CSBM are consistent with the structural differences identified in the *in vitro* assessments. The higher α-helix/β-sheet ratio, reduced β-sheet aggregation, and lower total Amide I area in PSBM indicate a more accessible and less compact protein matrix, features that likely translated into more efficient nutrient and energy absorption in vivo. Processing-induced matrix disruption would have enhanced the availability of intracellular energy sources, including residual lipids and associated organic matter, by minimizing physical entrapment within fibrous and proteinaceous structures (Milani et al., 2022).

Importantly, PSBM impact on digestion kinetics aligns with the physiological demands of broilers. Their short intestinal transit time (2 to 4 h) and high metabolic rate necessitate rapid nutrient availability (Mohammadigheisar and Kim, 2018; Ravindran, 2013). The markedly higher differences between PSBM and CSBM on *in vitro* N solubility and modest differences on SID of AA reflects the limitations of static digestion models. This discrepancy has also been noted in previous studies (Chen et al., 2019; Ton Nu et al., 2020), as *in vivo* systems incorporate dynamic regulatory mechanisms such as ileal brake feedback via peptide YY and adaptive pancreatic enzyme secretion, which are absent in *in vitro* models (Cummings and Overduin, 2007). Nevertheless, the high gastric *in vitro* solubility suggested that PSBM may be particularly beneficial in broilers, whose digestive systems are still maturing, especially in terms of pepsin and pancreatic proteases secretion during the critical first 14–21 days post-hatch (Ravindran and Reza Abdollahi, 2021). The SID data from 21-day-old broilers support the conclusion that PSBM processing enhances protein and amino acid digestibility, suggesting that PSBM could be a valuable component in starter feeds to capitalize on the rapid growth phase early in life.

Animal-derived proteins such as whey and casein are renowned for promoting muscle recovery and growth by rapidly delivering amino acids post-ingestion (Wilborn et al., 2013). Whey has high solubility and rapid gastric emptying, enabling rapid plasma availability of amino acids, while casein forms an acidic curd that delays its digestion and subsequent absorption (Campbell et al., 2007). Remarkably, PSBM exhibited digestion kinetics that emulate these animal proteins. Under acidic conditions, PSBM formed aggregates that mimic the coagulation behavior of casein, yet it avoids the prolonged gastric retention typically associated with casein, often an artifact of a static *in vitro* models challenged by abrupt pH shifts. This unique aggregation in PSBM is linked to the formation of intramolecular complexes and an increased prevalence of β-turn structures, which may transiently aggregate when hydrolyzed by pepsin. Although similar intermolecular aggregates in pulses have been shown to reduce digestibility and solubility (Yang et al., 2024), PSBM overcomes these limitations by achieving hydrolysis rates that approach those of animal proteins. Such enhanced digestibility is critical for broilers, whose rapid growth cycles (35–42 days) demand precisely synchronized AA absorption to optimize muscle protein synthesis. In essence, PSBM not only mitigates the inherent limitations of traditional plant proteins by providing a rapid and efficient nutrient release profile, but it also bridges the nutritional gap between plant and animal protein sources. By harmonizing the temporal dynamics of amino acid release with the high metabolic demands of broilers, PSBM represents a strategic innovation in feed formulation, one that can significantly enhance growth performance in intensive poultry production systems.

## CRediT authorship contribution statement

**Felix M. Njeri:** Writing – original draft, Software, Methodology, Formal analysis, Data curation, Conceptualization. **Emily Kim:** Writing – review & editing, Investigation, Data curation. **Youngji Rho:** Writing – review & editing, Investigation, Data curation. **Mai Anh Ton Nu:** Writing – review & editing, Methodology, Conceptualization. **Astrid Koppenol:** Writing – review & editing. **Hagen Schulze:** Writing – review & editing, Conceptualization. **Matthew Nosworthy:** Writing – review & editing. **Anna K. Shoveller:** Writing – review & editing. **Lee-Anne Huber:** Writing – review & editing. **Elijah G. Kiarie:** Writing – review & editing, Supervision, Funding acquisition, Conceptualization.

## Disclosures

The authors declare the following financial interests/personal relationships which may be considered as potential competing interests:

Elijah Kiarie reports financial support was provided by AB Agri Ltd. Elijah Kiarie reports a relationship with Wallenstein Feed and Supply Ltd that includes: funding grants. Elijah Kiarie reports a relationship with Natural Sciences and Engineering Research Council of Canada-Alliance Programs that includes: funding grants. Elijah Kiarie reports a relationship with Ontario Agri-Food Innovation Alliance, AB Agri Ltd that includes: funding grants. HS and MATN were employees of AB Agri Ltd during the experiment period. If there are other authors, they declare that they have no known competing financial interests or personal relationships that could have appeared to influence the work reported in this paper.
